# Stopping the Rot

**DOI:** 10.1371/journal.pbio.0020213

**Published:** 2004-07-13

**Authors:** Henry Nicholls

## Abstract

Phytophthora species blight potatoes and tomatoes, devastate soybean, rot cacao, and are the cause of sudden oak death. Understanding this versatile genus will be key to its control

In July 2000, the finger of blame for a mysterious mass killer of Californian oak trees came to rest on a previously undescribed plant pathogen. From the initial identification of Phytophthora ramorum, it took less than four years to produce a draft sequence of its genome, one of the fastest-ever discovery-to-sequence stories for a complex pathogen. This achievement was a United States initiative, facilitated by the injection of federal and state funding into Phytophthora research. But the US is not alone in the battle against this genus. Phytophthora species cause thousands of millions of dollars of damage to the world's commercial crops every year: they blight potatoes and tomatoes, devastate the lucrative soybean, and rot cacao, threatening the world's supply of chocolate (Figure 1). But for Sophien Kamoun, Associate Professor of Plant Pathology at Ohio State University (Wooster, Ohio, United States), these destructive organisms present an exciting opportunity. There are some 60 species of Phytophthora, but so little is known about the genus, he says, that there are things about its species we didn't even know we didn't know. ‘What I find really exciting,’ he says, ‘is discovering these unknown unknowns.’

**Figure 1:**
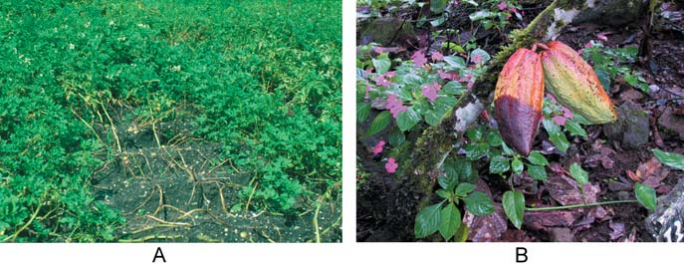
Phytophthora Infestations (A) Potato and (B) cacao pod. (Reproduced courtesy of Sophien Kamoun, Ohio State University [A], and Christopher J. Saunders and the USDA Agricultural Research Service [B].)

The most infamous of the Phytophthora pathogens is the potato late blight, P. infestans. It was this species that led to the Irish potato famine in the mid-1840s, which resulted in the death or displacement of millions. Today, P. infestans is estimated to cost potato and tomato farmers US$5,000,000,000 a year in lost revenue. The story for the soybean pathogen P. sojae is similar, causing loss of more than US$1,000,000,000 a year to soybean growers. In addition to the direct economic impact of these pathogens, introduced Phytophthora can cause severe damage to native flora. The most recent Phytophthora on the scene is P. ramorum, which has caused ‘sudden oak death’ (SOD) in tens of thousands of oak trees across the coastal counties of California, is now present in at least three other US states and is threatening to take on the native flora of the entire North American continent. It is also lurking in Europe, although apparently with less devastating consequences.

## Molecular Machinery

With this kind of impact, it's no surprise that money has poured into research on Phytophthora. This year, the US federal government will channel US$7,400,000 toward research into SOD. A major focus of this funding is genomics. Sequencing the genomes of several Phytophthora will help clarify the phylogeny and evolution of these enigmatic organisms (Box 1) and improve methods of detection and identification. Ultimately, however, sequencing should reveal the molecular tricks that this genus uses to subvert the defences of its plant hosts, allowing scientists to come up with new ways to combat these troublesome organisms.

The P. infestans sequencing initiative, coordinated by Kamoun, has recently completed a survey sequence of the genome that gives an initial understanding of how this organism is structured. Perhaps most striking is its size. ‘It's a huge genome,’ says Kamoun. At about 250 megabases (Mb), it's about twice the size of the Arabidopsis genome. His latest research, published in the *Journal of* *Biological Chemistry* (Tian et al. 2004), describes a P. infestans protease inhibitor—extracellular protease inhibitor 1 (EPI1)—that could be one of a unique class of suppressor proteins that Phytophthora deploy to infect and counteract host defences. The pathogen seems to upregulate the *epi1* gene during colonisation of its host. EPI1 inhibits plant apoplastic proteases—extracellular enzymes that are part of the host's defensive armoury that have evolved to prevent foreign proteins entering cells.

‘Based on its biological activity and expression pattern, EPI1 may function as a disease effector molecule and may play an important role in P. infestans colonisation of host apoplast,’ Kamoun and his colleagues report (Tian et al. 2004). If further research confirms this function for EPI1, then it will become one of just a handful of pathogen molecules that have been shown to suppress host plant defenses. A search in sequence databases for matching motifs reveals just one similar sequence in the entire bacterial and fungal kingdoms. However, apicomplexans like Toxoplasma gondii that transit through the mammalian digestive tract also appear to secrete protease inhibitors allied to EPI1. This similarity suggests an analogy between plant apoplasts and mammalian digestive tracts. Both environments are rich in proteases, but nevertheless are colonised by a variety of microbial pathogens. In the case of an apoplast, the pathogen is P. infestans, whilst in the mammalian gut, it's T. gondii—and although they are phylogenetically distant, these pathogens seem to have independently recruited similar secreted proteins to inhibit the defensive proteases produced by their hosts. Interestingly, whilst T. gondii inhibitors inhibit gut enzymes trypsin and chymotrypsin, EPI1 does not, suggesting that coevolution between the inhibitors and their target proteases may shape the specificity of these pathogenic enzymes.

Armed with this new insight into the molecular cunning of P. infestans, Kamoun hopes that it will be possible to come up with ways of slowing disease progression. Importantly, it looks likely that protease inhibitors such as EPI1 are present in other Phytophthora species. There are significant matches between the *epi1* gene sequence and motifs from at least five other closely related species. So any methods of blocking the action of protease inhibitors in P. infestans might also work against other Phytophthora. The protease inhibitors are one of Kamoun's ‘unknown unknowns’. ‘It's an example of something that we had absolutely no idea was in the genome,’ he says.

Even more advanced than the P. infestans genome project is an ongoing collaboration between the Virginia Bioinformatics Institute (Blacksburg, Virginia, United States) and the US Department of Energy's Joint Genome Project based in Walnut Creek, California. The focus here is the soybean pathogen P. sojae and the SOD pathogen P. ramorum, for which draft sequences are now complete (www.jgi.doe.gov/). The genomes are much smaller than that of the 250-Mb P. infestans—P. sojae is about 90 Mb and P. ramorum is just 55 Mb. This, in part, explains the comparative speed of these sequencing efforts. But another factor is undeniably the fear of the unknown P. ramorum, which in 2002 netted the Virginia–California initiative US$3,800,000 in federal funding to describe its genome. Ultimately, however, the sequence of one species will help to inform on the sequence of other related species.

Brett Tyler, Research Professor at the Virginia Bioinformatics Institute, is focusing on the molecular tools used by P. sojae to infect its host. He agrees with Kamoun that understanding this machinery is the way to devise new control measures that could give plants the upper hand in the evolutionary arms race against their Phytophthora pests. At present, most strategies to limit the damage caused by species like P. infestans and P. sojae rely on developing resistant cultivars by selective breeding of varieties with major resistance genes—single genes that can block a pathogen. However, Phytophthora seem able to find ways to overcome these efforts. ‘P. infestans is absolutely notorious for its ability to genetically change in response to a major resistance gene,’ says Tyler. ‘Typically major resistance genes in potato barely last a single season.’

Things look better for the soybean. New cultivars containing major resistance genes show resilience to P. sojae for five to ten years. However, this resistance is starting to break down, and breeders are running out of major resistance genes with which to conjure new varieties. The alternative, says Tyler, is quantitative or multigenic resistance, which relies on getting plants to express several resistance genes at once, each of which makes a small contribution to the plant's overall resistance. It should be much harder for P. sojae to evolve a new attack against this kind of robust defence. The search is also on for new genes that could be used to encourage quantitative resistance to host species. One particularly promising approach is to pit pathogen against host to see which genes are switched on. The host should upregulate genes that defend it against infection and the pathogen should upregulate genes that it needs to attack. The discovery that plants produce proteases and that Phytophthora have responded by secreting protease inhibitors to disable them is important if long-lasting solutions are to be found. ‘We just need to find a way to introduce new protein-degrading enzymes into the plant that the pathogen doesn't know how to block,’ says Tyler. ‘With these genomic tools we can really accelerate the pace at which we can evaluate different possible protective measures.’

## Epidemiology: Identifying the Culprit

An additional benefit of the abundance of genetic information is that species identification is becoming increasingly sophisticated. At a glance, Phytophthora can be mistaken for a fungus, so DNA profiling of isolates is crucial if species and strains of species are to be identified correctly so that action appropriate to each infection can be taken. Since 2000, Matteo Garbelotto, Adjunct Professor of Mycology and Forest Pathology at the University of California at Berkeley (Berkeley, California, United States), has spent a significant part of his working life tracking the spread of P. ramorum, the Phytophthora that has killed off vast tracts of oak trees in native Californian forest (Box 2).

DNA analysis has been crucial to confirm suspected cases of P. ramorum, and has now revealed that infections have reached at least three other US states. This spread is probably due to the movement of infected ornamentals like rhododendron (Rhododendron spp.) and viburnum (Viburnum spp.), which seem to act as carriers for the pathogen. ‘What we're seeing is a parallel to what has been happening throughout Europe, where the infection has basically moved using the commercial routes of the ornamental plant industry,’ Garbelotto says.

## P. ramorum in Europe

P. ramorum does not appear to have the same devastating consequences in Europe as it does in the US—at least not yet. It's not entirely clear why, but it could have something to do with the structure of the bark of different host species, suggests Garbelotto. ‘We normally see more infection where we have more corrugation, and that's because water accumulates in the fissures … where the zoospores have a chance to infect the bark.’ However, he notes, European beech (Fagus sylvatica) appears to be extremely susceptible to P. ramorum. ‘If it reaches areas where there are a lot of beeches, it could potentially mirror what's happening in California,’ he warns.

In Europe, symptoms characteristic of P. ramorum infection were first described on rhododendrons in The Netherlands in 1993. Once this was confirmed to be the same species as the pathogen responsible for Californian SOD, there was speculation that P. ramorum had either been introduced to the US from Europe or vice versa. However, in December 2002, it emerged that these two populations are of different mating types—A1 in Europe and A2 in the US. The latest research from Garbelotto and his colleagues, due to be published in Mycological Research (Ivors et al. 2004), supports this interpretation, demonstrating that although they belong to the same species, A1 and A2 are distinct lineages and have not exchanged genes for a long time. But last year, a batch of isolates from infected camellias (Camellia spp.) and rhododendrons in a nursery in Washington state showed that the A1 and A2 strains were living side by side. ‘That was a big surprise for us,’ recalls Garbelotto. ‘We had no knowledge at that point that both the European and the North American type could be present in the same nursery.’

## The Threat of Recombination

Hybridisation between different species of Phytophthora can produce a new species with different properties from those of either parent. One of the best cases comes from Europe, where a new Phytophthora emerged in 1993 that began to attack alder trees (Alnus spp.). Research carried out by scientists in the United Kingdom demonstrated that the alder Phytophthora was a product of a hybridisation event between P. cambivora and an unknown species similar to P. fragariae, neither of which attacks alder.

Given this propensity for Phytophthora species to hybridise and new phenotypes to emerge, there is legitimate concern that sexual recombination between the A1 and A2 mating types could produce something more devastating than either form. Within the controlled confines of his laboratory, Garbelotto has been exploring whether the two types of P. ramorum can get it together. Initial findings are that oospores—the product of sexual recombination—are being produced, although most of them abort before they reach maturity. However, 30% progress further, and (microscopically, at least) look like they could be functional. ‘They'll germinate,’ he predicts.

The threat that hybridisation could create a novel strain with a different host range is a concern that the UK is also taking seriously. In December 2003, an entirely new Phytophthora was isolated from two sites in England. Although the new species—currently referred to as Phytophthora taxon C sp. nov. (P. taxon C)— appears to cause relatively mild damage to its beech and rhododendron hosts, the UK's Department for Environment, Food and Rural Affairs (DEFRA) acknowledges that hybridisation of P. taxon C with P. ramorum could have serious consequences. ‘The potential for the pathogen to adapt further to its putative new environment intrinsically or via hybridisation is not known,’ note the authors of a DEFRA report on the mystery species (www.defra.gov.uk/planth/pra/forest.pdf). ‘Long-distance spread could easily occur through the movement of infected stock of rhododendron or beech and possibly other (as yet unknown) hosts,’ they warn.

**Figure 4:**
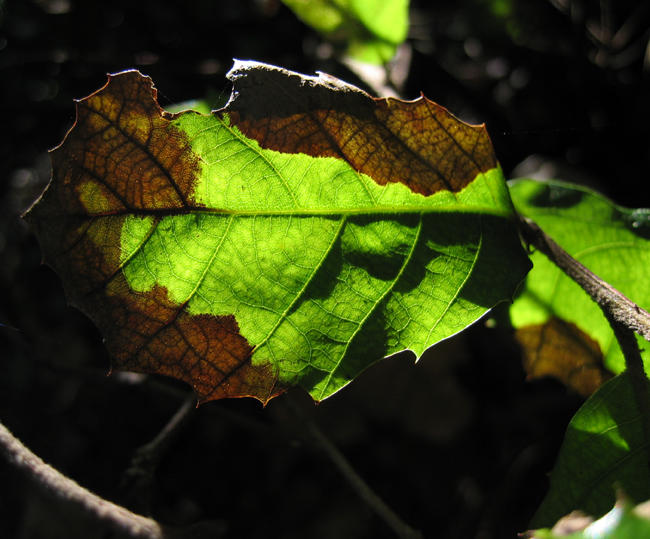
Infection of P. ramorum at a site in the UK DEFRA is monitoring closely for signs of hybridisation with P. taxon C sp. nov.

DEFRA is monitoring the situation closely (Figure 4). However, on the basis of a preliminary DNA analysis, P. taxon C and P. ramorum are only distantly related, making hybridisation unlikely, says Joan Webber, Head of Pathology at the UK government's Forestry Commission. The closest known relative of P. taxon C is P. boehmeriae, a pathogen that has been recorded on several species of tree in China and Australia, and on cotton in China and Greece, suggesting possible origins for the newly described species. But, says Webber, the sequence match between P. taxon C and P. boehmeriae is only 92%—not especially close. Much more evidence is needed to build a strong case for the origin of this new Phytophthora, she says.

## Origins

Indeed, it has taken more than 150 years to track down the geographical origin of the P. infestans strain that caused the Irish potato famine. Jean Beagle Ristaino of North Carolina State University (Raleigh, North Carolina, United States) is due to publish in Mycological Research an analysis of DNA extracted from diseased potato plants preserved from the nineteenth-century Irish epidemic (May and Ristaino 2004). It had long been suspected that the famine was caused by the Ib strain of P. infestans, which is presumed to have originated in Mexico. However, Ristaino's molecular evidence spotlights the Ia strain and traces its probable roots to the Andes. The infection could have found its way from South America to Europe and the US via exports of potato seed on steamships, she speculates.

This kind of forensic treatment is more than just interesting. Tracing a Phytophthora species to its site of origin could reveal what keeps them at bay in the areas where they are native, and might suggest new ways to manage them when they are introduced to a different ecosystem, says Garbelotto: ‘There's a huge amount of information that can be learned from understanding where they're coming from.’ So where do pathogens like P. ramorum originate? The best lead, Garbelotto says, is the ease with which it infects rhododendron. These ornamentals are natives of Asia, but there are only a few places where the climate would suit P. ramorum. The most promising, he suggests, are the Southern Himalayas, the Tibetan plateau, or Yunnan province in China. But these are big places, and Garbelotto has plenty on his plate in his battle against the Californian SOD. ‘I am not very hopeful that we'll ever be able to find out where it comes from,’ he says.

**Figure 2:**
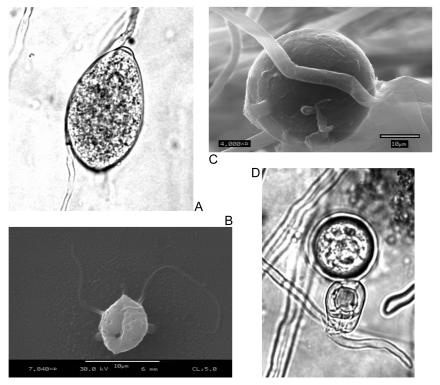
Reproductive Structures of the Phytophthora The asexual (A) sporangia, (B) zoospores, and (C) chlamydospores, and the sexual (D) oospores. (Reproduced courtesy of Matteo Garbelotto, UC Berkeley [A, D], and Edwin R. Florance, Lewis & Clark College [Portland, Oregon, United States] and the USDA Forest Service Pacific Southwest Research Station in Albany, California [B, C].)

**Figure 3:**
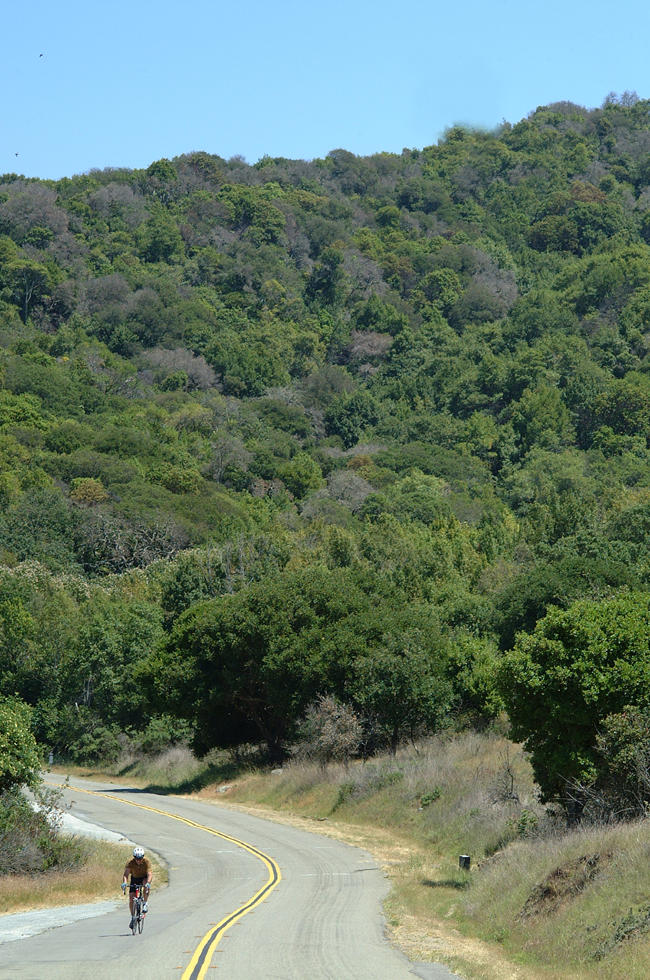
Coast Live Oaks Plagued by P. ramorum, Marin County, California (Reproduced courtesy of Matteo Garbelotto, UC Berkeley.)

**Box 1. A Closer Look at Phytophthora**

Phytophthora belong to the Kingdom Stramenophiles, so are most closely related to brown algae and diatoms. Their hyphal growth and variety of spores are morphologically and physiologically similar to fungi, for which they are occasionally mistaken, but their parasitic lifestyles have independent evolutionary origins, and therefore they have alternative mechanisms of pathogenicity. Within the class Oomycetes, which comprises all manner of heterotrophic blights, mildews, and molds, the Phytophthora—from the Greek for ‘plant destroyer’—is a pernicious genus, costing the world's farmers thousands of millions of dollars each year in control measures and lost yield. The majority of the 60 or so Phytophthora species that have been described are distinguished by their complex life histories, with both asexual and sexual phases and a bewildering array of associated reproductive structures. Sporangia are asexual spores that provide the pathogen with a short-lived mode of transmission. They are either broken off from the filamentous hyphae to become airborne as in the potato blight P. infestans (Figure 2A) or remain attached and divide into swimming zoospores following rains (Figure 2B). By contrast, chlamydospores are asexual structures that are adapted for longterm survival (Figure 2C). In the case of the Phytophthora causing ‘sudden oak death’ (P. ramorum) and forest dieback (P. cinnamomi), the chlamydospores play a crucial role, allowing the pathogen to survive from season to season. The formation of sexual structures is relatively rare, but oospores—the product of sexual recombination—occur in many species (Figure 2D). Some, such as P. sojae, are known as homothallic, with a single form that has both male and female reproductive structures, whilst others like P. ramorum are heterothallic and require two different mating types to meet for sexual recombination to occur.

**Box 2. Sudden Oak Death in California and Beyond**

In 1995, trees in oak forests in the coastal counties of California started to show a range of alarming and lethal symptoms. Since then, the disease has reached epidemic proportions, with tens of thousands of trees dying along approximately 300 km of the central Californian coastline (Figure 3). By 2000, the pathogen responsible had been identified as P. ramorum, and it is now clear that this pest has an extremely broad host range that extends to almost all woody plant species in the coastal forests of California. Many oak hosts suffer lethal branch or stem infections, whilst non-oak hosts only carry mild leaf or twig infections. The species hardest hit include tanoak (Lithocarpus densiflora) and the true oaks coast live oak (Quercus agrifolia), California black oak (Quercus kellogii) and Shreve's oak (Quercus parvula var. shrevei). These species develop large wounds or cankers in their woody tissue, which disrupt physiology and in extreme cases lead to death in a matter of months.

In non-oak species, P. ramorum does not appear to cause the same damage, leaving hosts like the Californian bay laurel (Umbellularia californica) and rhododendrons (Rhododendron spp.) with the relatively mild symptoms of leaf blight and occasional branch dieback. However, these non-oak hosts probably act as reservoirs of disease and may even help it to spread. The sporangia and chlamydospores that are thought to be the main asexual propagules of the pathogen (Box 1) are readily produced on the foliage of such non-oak species, but do not seem to appear on the bark of most of the infected oak hosts.

In March 2004, P. ramorum was found at a large wholesale horticultural nursery in Los Angeles County. The California Department of Food and Agriculture, the US Department of Agriculture's Animal and Plant Health Inspection Service, and state agriculture departments around the country are tracing all plants shipped from this nursery over the past year in an effort to identify and destroy any infected material, and hence prevent any further spread of the disease. However, the rot has already set in at nurseries in three states outside California: Oregon, Washington, and Florida. A National SOD Nursery Survey is underway to try to assess how far the disease might have spread.

## Abbreviations

DEFRA: United Kingdom Department for Environment
EPI1: extracellular protease inhibitor 1
Mb: megabase(s)
SOD: sudden oak death

## References

[pbio-0020213-Brasier1] Brasier CM, Cooke DEL, Duncan JM (1999). Origin of a new Phytophthora pathogen through interspecific hybridization. Proc Natl Acad Sci U S A.

[pbio-0020213-DEFRA1] DEFRA (2004). Pest risk analysis. Phytophthora taxon C sp. nov. www.defra.gov.uk/planth/pra/forest.pdf.

[pbio-0020213-Erwin1] Erwin DC, Ribiero OK (1996). Phytophthora diseases worldwide.

[pbio-0020213-Garbelotto1] Garbelotto M, Davidson JM, Ivors K, Maloney P, Huberli D (2003). Non-oak native plants are the main hosts for the sudden oak death pathogen in California. Calif Agr.

[pbio-0020213-Huitema1] Huitema E, Bos JIB, Tian M, Win J, Waugh ME (2004). Linking sequence to phenotype in Phytophthora–plant interactions. Trends Microbiol.

[pbio-0020213-Ivors1] Ivors KL, Hayden KJ, Bonats PJM, Rizzo DM, Garbelotto M (2004). AFLP and phylogenetic analyses of North American and European populations of Phytophthora ramorum. Mycol Res.

[pbio-0020213-May1] May KJ, Ristaino JB (2004). Identity of the mitochondrial DNA haplotype(s) of Phytophthora infestans in historical specimens from the Irish potato famine. Mycol Res.

[pbio-0020213-Rizzo1] Rizzo DM, Garbelotto M (2003). Sudden oak death: Endangering California and Oregon forest ecosystems. Front Ecol Environ.

[pbio-0020213-Tian1] Tian M, Huitema E, da Cunha L, Torto T, Kamoun S (2004). A Kazal-like extracellular serine protease inhibitor from Phytophthora infestans targets the tomato pathogenesis-related protease P69B. J Biol Chem.

[pbio-0020213-Tyler1] Tyler BM (2001). Genetics and genomics of the oomycete–host interaction. Trends Genet.

[pbio-0020213-Tyler2] Tyler BM (2002). Molecular basis of recognition between Phytophthora species and their hosts. Annu Rev Phytopathol.

[pbio-0020213-Werres1] Werres S, Zielke B (2003). First studies on the pairing of Phytophthora ramorum. J Plant Dis Prot.

